# The multifaceted expression of the gastrin releasing peptide receptor in prostate cancer

**DOI:** 10.1038/s41598-026-64701-7

**Published:** 2026-08-12

**Authors:** Clément Morgat, Mégane Le Quang, Tyrillshall S. T. Damiana, Wytske M. van Weerden, Gaëtan Macgrogan, Valérie Vélasco, Mokrane Yacoub, Marine Gross-Goupil, Elif Hindié, Simone U. Dalm

**Affiliations:** 1 Department of Nuclear Medicine & Radiopharmacy, CHU de Bordeaux, Bordeaux, F-33000 France; 2 University of Bordeaux, CNRS, EPHE, INCIA, UMR 5287, Bordeaux, F-33000 France; 3 Department of Pathology, Bordeaux University Hospital, Bordeaux, France; 4 Department of Radiology & Nuclear Medicine, Erasmus Medical Center, Rotterdam, 3015 GD The Netherlands; 5 Department of Urology, Erasmus Medical Center, Rotterdam, 3015 GD The Netherlands; 6 Surgical Pathology Unit, Department of Biopathology, Institut Bergonié, Bordeaux, F-33076 France; 7 ACTION U1218, INSERM, Bordeaux, F-33076 France; 8 Department of Medical Oncology, University Hospital of Bordeaux, Bordeaux, F-33076 France; 9 Institut Universitaire de France, IUF, Paris, F-75000 France

**Keywords:** GRPR, Prostate cancer, Neuroendocrine prostate cancer, Targeted radionuclide therapy, PSMA, Cancer, Oncology

## Abstract

Gastrin releasing peptide receptor (GRPR) gained interest for targeted radionuclide therapy (TRT) of prostate cancer (PCa). However, its expression in advanced disease remains understudied. We correlated clinical, biological, molecular and pathological characteristics, with GRPR expression in PCa patient-derived xenograft (PDX) models, and compared it with prostate specific membrane antigen (PSMA) expression. GRPR expression was studied on tissue microarrays of 107 PDXs using immunohistochemistry. Seventy-six samples were GRPR positive. There were no differences in GRPR expression between primary and metastatic lesions, and androgen-naïve, androgen sensitive and castration resistant samples. High GRPR expression was frequent in androgen receptor (AR) positive tumors, high PSMA-expressing tumors, tumors without phosphatase and tensin homolog (PTEN) loss, and vimentin negative tumors. AR-positivity was the most important predictor of high GRPR expression. In PSMA negative tumors, GRPR expression was high in chromogranin positive tumors, tumors without PTEN loss, in absence of retinoblastoma1-gene mutation, and high vimentin-expressing tumors. Among the neuroendocrine PCa’s (NEPC), 14/20 were GRPR and 3/20 were PSMA positive. All PSMA positive tumors were GRPR positive. Among metastatic NEPC samples, 9/10 and 1/10 were GRPR and PSMA positive, respectively. Based on the aforementioned, we conclude that GRPR is a relevant target for advanced PCa. Moreover, the selective GRPR expression in NEPC opens new avenues for GRPR-TRT.

**Supplementary Information:**

The online version contains supplementary material available at https://doi.org/10.1038/s41598-026-64701-7.

## Introduction

Prostate cancer (PCa), one of the most commonly diagnosed malignancies in men worldwide, presents significant variability in its clinical profile^1^. Its progression is influenced by molecular disruptions across several key regulatory pathways, including apoptosis, androgen receptor (AR) signaling, cell cycle regulation, adhesion, signal transduction, and angiogenesis^2^.

Despite the advancements made over the years with regards to understanding PCa biology and the development of novel methods to manage the disease, clinical outcomes remain suboptimal for many PCa patients. This is particularly true for patients with advanced or metastatic disease, although novel therapeutics are becoming available^3^. Despite established therapeutic benefit from prostate specific membrane antigen (PSMA)-targeting radionuclide therapy (e.g., with [^177^Lu]Lu-PSMA-617 or [^225^Ac]Ac-PSMA-617), responses are rarely durable. Moreover, PSMA-targeting is not suitable for patients with low PSMA-expressing tumors^4^. Treatment-emergent neuroendocrine PCa, in particular, is well known to be associated with a decrease in PSMA expression^5^. Therefore, alternative targets for targeted radionuclide therapy (TRT) of PCa are being investigated, including delta-like ligand 3 (DLL3)^6^, CUB domain-containing protein 1 (CDCP1)^7^, membrane cofactor protein (CD46)^8^, gastrin releasing peptide receptor (GRPR) ^9^, and others.

GRPR, is a member of the bombesin receptor family which is expressed at low to negligible levels in normal tissues but is upregulated in many malignant conditions, including PCa, breast cancer, small cell and non-small cell lung cancer, and gastrointestinal stromal tumors, and others, making it an attractive molecular target for diagnostic and therapeutic purposes, including the aforementioned TRT^10^. In PCa, however, factors driving GRPR expression are not well understood. It is well established that GRPR is expressed at high levels in early stages of PCa^11,12^. Recent reports indicate that GRPR expression can be maintained in advanced PCa and that GRPR positive tumors might have a shorter progression free survival (PFS)^13^. Moreover, seminal studies have shown high GRPR expression in neuroendocrine PCa (NEPC) while PSMA expression was weak. Therefore, GRPR might hold a two-faced expression which needs to be understood. In this study we investigated GRPR expression on tissue microarrays (TMAs) of over one hundred authenticated PCa patient-derived xenografts (PDXs), mainly advanced tumors, using immunohistochemistry (IHC). We also aimed to correlate clinical, biological, molecular and pathological characteristics, with GRPR expression, and in comparison to PSMA expression. PDX models are a reliable preclinical trial platform, retaining key features of the original tumor and thus they accelerate drug discovery for faster translation to clinical trials^14^.

## Materials and methods

### PCa TMAs

In this study TMAs containing PDX material were assessed. No animals, human participants or human tissue samples were used in the current study. Thus, no ethical approval was required. Nevertheless, the university of Bordeaux holds a license for the hold and use of human tissue samples (ARDC-2023-5565).

The PCa PDX TMAs provide a robust platform for translational research and therapeutic evaluation and were subjected to GRPR-IHC. The experimental protocol was approved by the coordinating center (MD Anderson Cancer Centre, University of Texas, USA). Characteristics of the samples (total *n* = 107) are described in Table 1. Cut-off of IHC against molecular markers were those used from the original publication^15^. Of note the cut-off used for PSMA-positivity was 20/200^15^. The current study was performed in accordance with REMARK guidelines.

**Table 1:** Study population

		n
**Origin of the samples**		
Biopsy		20
Radical prostatectomy		20
Cystoprostatectomy		13
Autopsy		24
Other		22
Not specified		8
**Anatomic description**		
Primary tumor		31
Local extension (ureter, rectum, prostate/ bladder neck)		13
Metastatic disease		56
Not specified		7
**Histology**		
Adenocarcinoma		79
Adenocarcinoma with neuroendocrine differentiation		20 (6 de novo and 14 under treatment)
Not specified		8
**ISUP score**		
≤6		3
7		11
8-10		37
Not specified		
**Tumor site**		
Prostate		41
Bone		16
Lymph nodes		11
Liver		12
Other		19
Not specified		8
**Clinical state**		
Androgen-naïve		19
Androgen responsive		13
Castration resistant		60
Not specified		15
**Previous therapy**		
Treatment-naïve		19
Hormone therapy		6
Hormone therapy + chemotherapy		21
Hormone therapy + chemotherapy + radiation therapy		13
Hormone therapy + radiation therapy		3
Other		3
Not specified		42
**Molecular markers**		
AR	-	37
	+	70
Chromogranin A	-	87
	+	20
CYP17A1	-	105
	+	2
ERG	-	84
	+	23
FoxA1	-	82
	+	25
Ki-67	-	0
	+	100
p53	-	29
	+	78
pAKT	-	51
	+	56
PSMA	-	49
	+	58
PTEN	-	68
	+	39
Rb1 gene mutation	-	23
	+	84
Vimentin	-	56
	+	51

ISUP=International Society of Urological Pathology, AR=androgen receptor, CYP17A1=cytochrome P450 family 17 subfamily A member 1, ERG=ETS related gene, FoxA1=forkhead box protein A1, p53=tumor protein p53, pAKT=phosphorylated AKT, PSMA=prostate specific membrane antigen, PTEN=phosphatase and tensin homolog, Rb=retinoblastoma.

### Origin of TMAs

The studied TMAs were previously generated by MD Anderson in the context of the MOVEMBER GLOBAL ACTION PLAN PROSTATE CANCER - PATIENT DERIVED XENOGRAFTS (PDX) TMAs^16^. For this, MD Anderson received PCa PDX material from multiple institutions. Each institution completed ethical compliance through a signed material transfer agreement before distributing the samples for TMA generation. The following institutions contributed: Centre hospitalier de l’Université de Montréal (CHUM), Johns Hopkins Hospital (JHU), Helsinki University Hospital (HUS), Institute of Biomedicine, University of Turku and Turku University Hospital (TYKS), Oslo University Hospital (OUH), University of Mississippi Baptist Medical Centre (UMBMC), Greater Los Angeles VA Healthcare System, Durham VA Health Care System, Emory University, and the University of Washington. All the aforementioned centers received permission from their ethical boards for the use of the material and the study was conducted in accordance with ethical guidelines. This includes the following ethical boards: Comité d’éthique de la recherche du CHUM (CE.14.128), the Institutional Review Board of the: JHU School of Medicine’s, the UMBMC, the VA Greater Los Angeles (Department of Veterans Affairs PCC #2015–040408), the Ethics Committee of Hospital District of Helsinki and Uusimaa (84/13/03/00/2014; § 3 30.01.2015), the Hospital District of Southwest Finland (number T206/2014) National Supervisory Authority for Welfare and Health for HUS and TYKS (VALVIRA, 8008/06.01.03/2014), the Regional Committees for Medical and Health Research Ethics for OUH (REC 2013/1713), and Center for Healthcare Ethics for Cedar Sinai (Pro00033387 and Pro00020577). Written patient informed consents were obtained (CHUM CE12.216, HUS, TYKS, and University of Washington, Cedar Sinai) or waivers were granted (JHU, OUH, both VA Health Care System and UMBMC) by the ethical review boards.

### GRPR-IHC and scoring

GRPR-IHC was performed as previously described^17,18^. The polyclonal rabbit antibody TA 316,872 (Origene Technologies Rockville, Maryland, USA) was used at 1/1400 dilution. This antibody has been previously validated using both western blotting and IHC^17,18^. Controls include irrelevant immunoglobulin G (Rabbit Polyclonal Negative Control Ig, ready to use, Roche Diagnostics, first negative control), as well as omission of the primary antibody (second negative control). The IHC was quantified following the h-score in which both the percentage of tumor cells stained (0–100) and the staining intensity (0–3) are considered, yielding a score that can range from 0 to 300. This cut-off corresponds to an immunoreactive score (IRS) of 6. In our previous studies^17,18,^ we used an IRS of 4 as cut-off but this failed to correlate to in vivo positron emission tomography (PET) scans^19^.

### Data analysis

To understand the expression of GRPR in PCa, continuous data were used. Samples were divided based on anatomic description (primary tumor, local extension, metastasis), histology (adenocarcinomas with and without neuroendocrine differentiation, etc.), grading (International Society of Urological Pathology (ISUP) score), clinical state (androgen-naïve, androgen responsive, castration resistant), tumor site (prostate, bones, lymph nodes, etc.) and previous therapy (none, hormone therapy, chemotherapy, etc.), and the mean GRPR h-score for each group was determined.

For association with other parameters, data on GRPR expression were dichotomized into 2 groups: low GRPR “GRPR negative” (h-score < 100) and high GRPR “GRPR positive” (≥ 100). Associations between GRPR expression and chromogranin, cytochrome P450 family 17 subfamily A member 1 (CYP17A1), ETS related gene (ERG), forkhead box protein A1 (FoxA1), the marker of proliferation Ki-67, tumor protein p53 (p53), phosphorylated AKT (pAKT), PSMA, phosphatase and tensin homolog (PTEN), retinoblastoma (Rb), and vimentin were studied. Differences between categorized variables were assessed with the chi² test or Fischer’s test when appropriate. Differences in more than two groups were assessed using non-parametric one-way ANOVA (Kruskal-Wallis test) and results were corrected for multiple comparison (Tukey’s method). P values were two-sided and *p* < 0.05 was considered statistically significant.

Factors that were predictive for high GRPR expression were investigated by univariate and multivariate analysis using a logistic regression. Variables that were significant in the univariate analysis were introduced in the multivariate analysis. Odds ratios (OR) and their 95% confidence intervals (95% CIs) were reported. Jamovi software (v2.6.26) was used for statistical analysis.

## Results

### GRPR expression

**Fig. 1:**
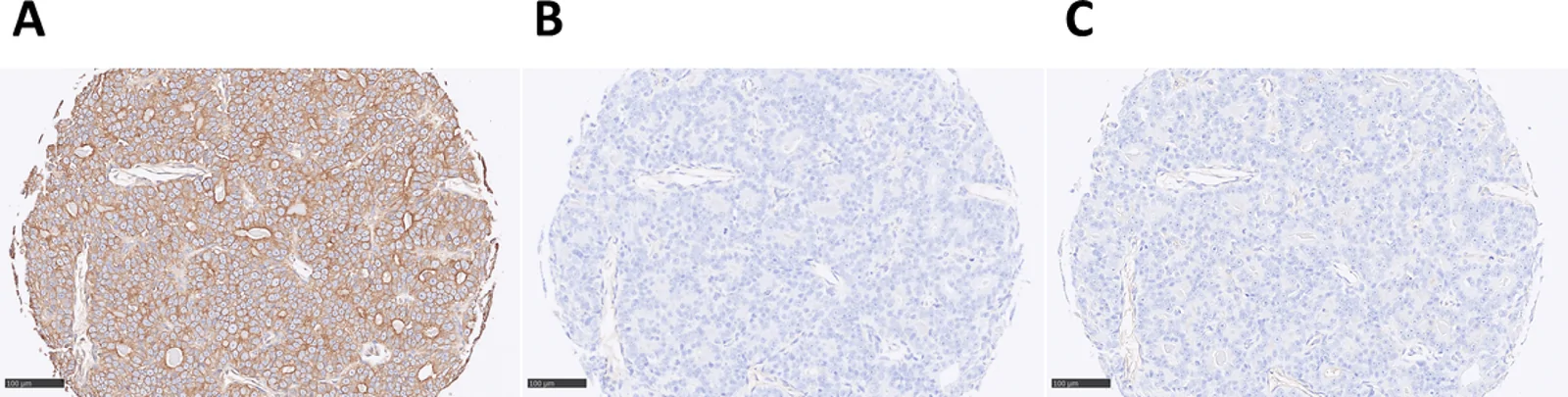
Representative gastrin releasing peptide receptor (GRPR) immunohistochemistry (**A**) with membranous staining. The irrelevant immunoglobulin G control (**B**) and omission of the primary antibody (**C**) yielded samples with no GRPR staining.

GRPR-IHC showed a membranous staining (Fig. 1A) with weak or no signal being observed when using the irrelevant immunoglobulin G (Fig. 1B) or when omitting the primary antibody (Fig. 1C). The mean GRPR expression was 161.5/300. Seventy-six samples (71.0%) were GRPR positive (h-score ≥ 100).

**Fig. 2:**
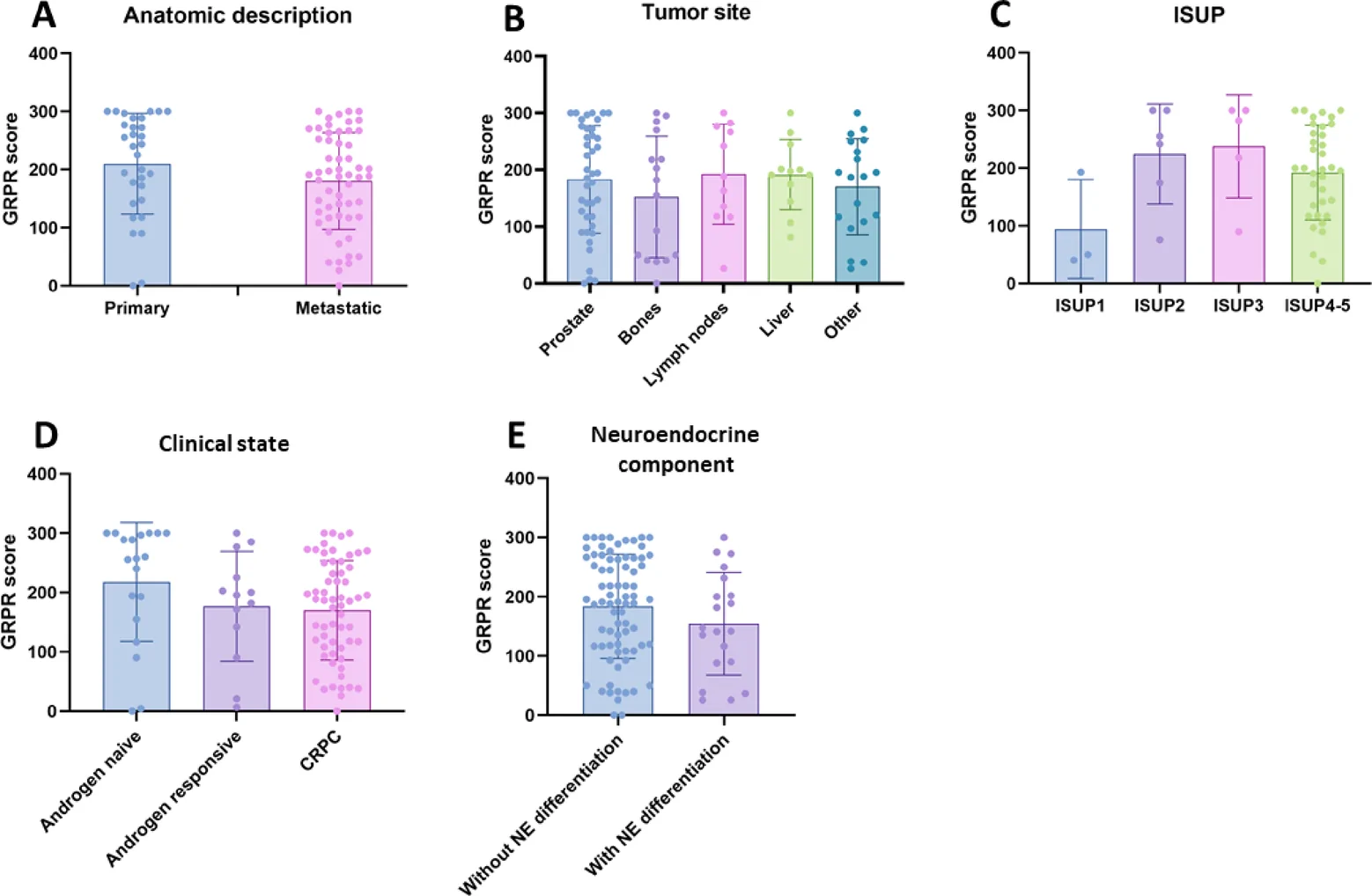
Gastrin releasing peptide receptor expression according to clinical and pathological parameters. ISUP=International Society of Urological Pathology score, CRPC=castration resistant prostate cancer, NE = Neuroendocrine.

Association of GRPR expression with clinical parameters showed no significant differences in GRPR expression between primary and unpaired metastatic samples (Fig. 2A, *p* = 0.3216), no significant differences in relation to tumor sites (Fig. 2B, adjusted P-values > 0.05), no significant differences in ISUP groups (Fig. 2C, adjusted P-values > 0.05), no significant differences between androgen-naïve, androgen sensitive and castration resistant PCa (CRPC) (Fig. 2D *p* = 0.061, ANOVA; adjusted P-value androgen-naïve vs. androgen sensitive is 0.4756, adjusted P-value androgen-naïve vs. CRPC is 0.0545; adjusted P-value androgen sensitive vs. CRPC > 0.999), and no significant differences in GRPR expression in samples with and without neuroendocrine differentiation (Fig. 2E, *p* = 0.1679).

Next, a contingency analysis was performed between GRPR and molecular markers to better understand the molecular profile of the GRPR positive tumors (Table 2). High GRPR expression was more frequent in AR positive tumors (84.06% vs. 44.74%; *p* < 0.001), high PSMA-expressing tumors (85.96% vs. 52.94%, *p* < 0.001), tumors without PTEN loss (89.74% vs. 59.42%, *p* = 0.003), and vimentin negative tumors (77.97% vs. 58.82%, *p* = 0.015).

**Table 2:** Distribution of low and high gastrin releasing peptide receptor expression

	N (%)	GRPR- (n (%))	GRPR+ (n (%))	***p***
AR-	37 (100%)	20 (55.26%)	17 (44.74%)	**< 0.001**
AR+	70 (100%)	11 (15.94%)	59 (84.06%)	
Chromogranin-	87 (100%)	29 (32.95%)	58 (67.05%)	0.283
Chromogranin+	20 (100%)	3 (15.79%)	17 (84.21%)	
ERG-	84 (100%)	25 (30.26%)	59 (69.74%)	0.139
ERG+	23 (100%)	2 (10.71%)	21 (89.29%)	
FoxA1-	82 (100%)	29 (35.80%)	53 (64.20%)	0.217
FoxA1+	25 (100%)	3 (11.11%)	22 (88.89%)	
p53-	29 (100%)	4 (14.81%)	25 (85.19%)	0.204
p53+	78 (100%)	26 (33.77%)	52 (66.23%)	
pAKT-	51 (100%)	19 (37.50%)	32 (62.50%)	0.113
pAKT+	56 (100%)	11 (20.34%)	45 (79.66%)	
PSMA-	49 (100%)	23 (47.06%)	26 (52.94%)	**< 0.001**
PSMA+	58 (100%)	8 (14.04%)	50 (85.96%)	
PTEN-	68 (100%)	28 (40.58%)	40 (59.42%)	**0.003**
PTEN+	39 (100%)	4 (10.26%)	35 (89.74%)	
Rb-	23 (100%)	5 (22.73%)	18 (77.27%)	0.334
Rb+	84 (100%)	26 (31.40%)	58 (68.60%)	
Vimentin-	56 (100%)	12 (22.03%)	44 (77.97%)	**0.015**
Vimentin+	51 (100%)	21 (41.18%)	30 (58.82%)	

GRPR=gastrin releasing peptide receptor, AR=androgen receptor, ERG = ETS related gene, FoxA1 = forkhead box protein A1, p53 = tumor protein p53, pAKT=phosphorylated AKT, PSMA=prostate specific membrane antigen, PTEN=phosphatase and tensin homolog, Rb=retinoblastoma. Data for CYP17A1 (cytochrome P450 family 17 subfamily A member 1) and Ki-67 are not included because of the low number of positive (2 for CYP17A1) or negative (0 for Ki-67) samples. Significance is indicated in bold.

Predictors of GRPR expression were then assessed using univariate and multivariate analysis (Table 3). AR-positivity was observed to be the most important predictor (OR, 3.1662; 95%CI [1.0730–11.483], *p* = 0.04) in the multivariate analysis. PTEN-positivity (absence of PTEN loss) was also a significant predictor (OR, 2.9468 [1.1412–9.226], *p* = 0.02).

**Table 3:** Univariate and multivariate analysis of high gastrin releasing peptide receptor expression

	Univariate analysis					Multivariate analysis	
	N (%))	GRPR- (n (%))	GRPR+ (n (%))	***p***	Odds ratio [95% CI]	***p***	Odds ratio [95% CI]
AR-	37 (100%)	21 (56.80%)	16 (43.20%)	**< 0.001**	1	**0.04**	1
AR+	70 (100%)	11 (15.70%)	59 (84.30%)		7.040 [2.8194–15.578]		3.1662 [1.0730–11.483]
Chromogranin-	87 (100%)	28 (32.20%)	59 (67.80%)	0.283	1	Not retained	
Chromogranin+	20 (100%)	4 (20.0%)	16 (80.0%)		1.898 [0.5808–6.205]		
CYP17A1-	105 (100%)	31 (29.50%)	74 (70.50%)	0.531	1	Not retained	
CYP17A1+	2 (100%)	1 (50.0%)	1 (50.0%)		0.419 [0.0254–6.91]		
ERG-	84 (100%)	28 (33.30%)	66 (66.70%)	0.139	1	Not retained	
ERG+	23 (100%)	4 (17.40%)	19 (82.60%)		2.375 [0.7373–7.65]		
FoxA1-	82 (100%)	27 (32.90%)	55 (67.10%)	0.217	1	Not retained	
FoxA1+	25 (100%)	5 (20.00%)	20 (80.00%)		1.964 [0.6650–5.799]		
p53-	29 (100%)	6 (20.70%)	23 (69.30%)	0.204	1	Not retained	
p53+	78 (100%)	26 (33.30%)	52 (66.70%)		0.522 [0.189–1.439]		
pAKT-	51 (100%)	19 (37.30%)	32 (62.70%)	0.113	1	Not retained	
pAKT+	56 (100%)	13 (23.20%)	43 (76.80%)		1.964 [0.847–4.553]		
PSMA-	49 (100%)	24 (49.00%)	25 (51.00%)	**< 0.001**	1	0.255	
PSMA+	58 (100%)	8 (13.80%)	50 (86.20%)		6.000 [2.3599–15.255]		
PTEN-	68 (100%)	27 (39.70%)	41 (60.30%)	**0.003**	1	**0.023**	1
PTEN+	39 (100%)	5 (12.80%)	34 (87.20%)		4.478 [1.5561–12.887]		2.9468 [1.1412–9.226]
Rb-	23 (100%)	5 (21.70%)	18 (78.30%)	0.334	1		
Rb+	84 (100%)	27 (32.10%)	57 (67.90%)		0.586 [0.197–1.747]		
Vimentin-	56 (100%)	11 (19.60%)	45 (80.40%)	**0.015**	1	0.652	
Vimentin+	51 (100%)	21 (41.20%)	30 (58.80%)		0.349 [0.147–0.828]		

GRPR=gastrin releasing peptide receptor, AR=androgen receptor, CYP17A=cytochrome P450 family 17 subfamily A member 1, ERG = ETS related gene, FoxA1 = forkhead box protein A1, p53 = tumor protein p53, pAKT=phosphorylated AKT, PSMA=prostate specific membrane antigen, PTEN=phosphatase and tensin homolog, Rb=retinoblastoma. Significance is indicated in bold.

### GRPR expression in low PSMA-expressing tumors

Considering the interest of PSMA-targeting for TRT of PCa, a PSMA-stratified analysis was performed (Table 4). Among the 49 PSMA negative samples, high GRPR expression was found in chromogranin positive tumors (76.9% vs. 41.7%, *p* = 0.029), tumors without PTEN loss (90.9% vs. 39.5%; *p* = 0.003), in absence of RB1-gene mutation (73.7% vs. 36.7%, *p* = 0.012), and in tumors with high vimentin expression (70.6% vs. 40.6%, *p* = 0.046). Predictors of high GRPR expression were also assessed in the low PSMA-expressing population (Table 5). Absence of PTEN loss was the most important predictor (OR = 14.146; 95%CI [1.5168–131.94], *p* = 0.02).

**Table 4:** Distribution of low and high gastrin releasing peptide receptor expression in prostate specific membrane negative samples.

	N (%)	GRPR- (n (%))	GRPR+ (n (%))	***p***
AR-	35 (100%)	20 (57.1%)	15 (42.9%)	0.071
AR+	14 (100%)	4 (28.6%)	10 (71.4%)	
Chromogranin-	36 (100%)	21 (58.3%)	15 (41.7%)	**0.029**
Chromogranin+	13 (100%)	3 (23.1%)	10 (76.9%)	
CYP17A1-	48 (100%)	24 (50.0%)	24 (50.0%)	0.322
CYP17A1+	1 (100%)	0 (0.0%)	1 (100.0%)	
ERG-	45 (100%)	22 (48.9%)	23 (51.1%)	0.966
ERG+	4 (100%)	2 (50.0%)	2 (50.0%)	
FoxA1-	36 (100%)	20 (55.6%)	16 (44.4%)	0.125
FoxA1+	13 (100%)	4 (30.8%)	9 (69.2%)	
p53-	9 (100%)	4 (47.5%)	5 (52.5%)	0.662
p53+	40 (100%)	22 (55.6%)	18 (44.4%)	
pAKT-	27 (100%)	10 (36.4%)	17 (63.6%)	0.111
pAKT+	22 (100%)	13 (59.3%)	9 (40.7%)	
PTEN-	38 (100%)	23 (60.5%)	15 (39.5%)	**0.003**
PTEN+	11 (100%)	1 (9.1%)	10 (90.9%)	
Rb-	19 (100%)	5 (26.3%)	14 (73.7%)	**0.012**
Rb+	30 (100%)	19 (63.3%)	11 (36.7%)	
Vimentin-	17 (100%)	10 (59.4%)	7 (40.6%)	**0.046**
Vimentin+	32 (100%)	9 (29.4%)	23 (70.6%)	

GRPR=gastrin releasing peptide receptor, AR=androgen receptor, CYP17A1 = cytochrome P450 family 17 subfamily A member 1, ERG = ETS related gene, FoxA1 = forkhead box protein A1, p53 = tumor protein p53, pAKT=phosphorylated AKT, PSMA=prostate specific membrane antigen, PTEN=phosphatase and tensin homolog, Rb=retinoblastoma. No statistical test was performed on Ki-67 because all samples were positive. Significance is indicated in bold.

**Table 5:** Univariate and multivariate analysis of high gastrin releasing peptide receptor expression in low prostate specific membrane expressing samples

	Univariate analysis					Multivariate analysis	
	N (%)	GRPR- (n (%))	GRPR+ (n (%))	***p***	Odds ratio [95% CI]	***p***	Odds ratio [95% CI]
AR-	35 (100%)	20 (57.1%)	15 (42.9%)	0.078	1	Not retained	
AR+	14 (100%)	4 (28.6%)	10 (71.4%)		3.333 [0.874–12.72]		
Chromogranin-	36 (100%)	21 (58.3%)	15 (41.7%)	**0.037**	1	0.795	
Chromogranin+	13 (100%)	3 (23.1%)	10 (76.9%)		4.667 [1.0942–19.90]		
CYP17A1-	48 (100%)	24 (50.0%)	24 (50.0%)	0.991	1	Not retained	
CYP17A1+	1 (100%)	0 (0%)	1 (100%)		5.76E-6 [0.0 - inf]		
ERG-	45 (100%)	22 (48.9%)	23 (51.1%)	0.966	1	Not retained	
ERG+	4 (100%)	2 (50.0%)	2 (50.0%)		1.045 [0.135–8.08]		
FoxA1-	36 (100%)	20 (55.6%)	16 (44.4%)	0.133	1	Not retained	
FoxA1+	13 (100%)	4 (30.8%)	9 (69.2%)		0.356 [0.0923–1.37]		
p53-	9 (100%)	5 (55.6%)	4 (44.4%)	0.663	1	Not retained	
p53+	40 (100%)	19 (47.5%)	21 (52.5%)		1.382 [0.323–5.91]		
pAKT-	27 (100%)	16 (59.3%)	11 (40.7%)	0.114	1	Not retained	
pAKT+	22 (100%)	8 (36.4%)	14 (63.6%)		2.545 [0.799–8.11]		
PTEN-	38 (100%)	23 (60.5%)	15 (39.5%)	**0.013**	1	**0.020**	1
PTEN+	11 (100%)	1 (9.1%)	10 (90.9%)		15.333 [1.7760–132.385]		14.146 [1.5168–131.94]
Rb-	19 (100%)	5 (26.3%)	14 (73.7%)	**0.014**	1	0.146	
Rb+	30 (100%)	19 (63.3%)	11 (36.7%)		4.836 [1.368–17.094]		
Vimentin-	17 (100%)	5 (29.4%)	12 (70.6%)	0.051	1	Not retained	
Vimentin+	32 (100%)	19 (59.4%)	13 (40.6%)		0.285 [0.0809–1.00]		

GRPR=gastrin releasing peptide receptor, AR=androgen receptor, CYP17A1 = cytochrome P450 family 17 subfamily A member 1, ERG = ETS related gene, FoxA1 = forkhead box protein A1, p53 = tumor protein p53, pAKT=phosphorylated AKT, PSMA=prostate specific membrane antigen, PTEN=phosphatase and tensin homolog, Rb=retinoblastoma. Significance is indicated in bold.protein p53, pAKT=phosphorylated AKT, PSMA=prostate specific membrane antigen, PTEN=phosphatase and tensin homolog, Rb=retinoblastoma. Significance is indicated in bold.

### GRPR expression in NEPC

**Fig. 3:**
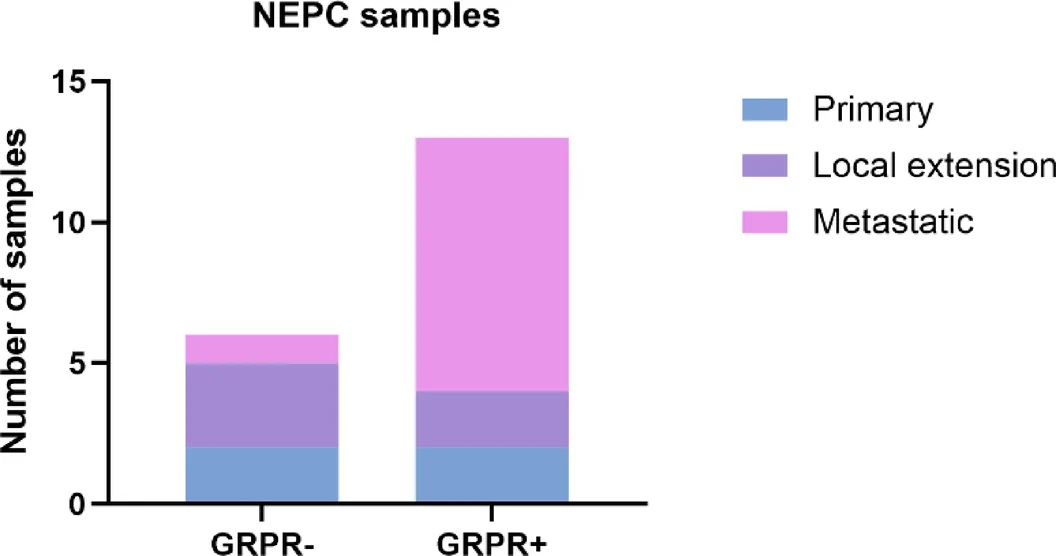
Distribution of low and high gastrin releasing peptide receptor (GRPR) expression in neuroendocrine prostate cancer (NEPC) samples. Data is missing for one sample.

High GRPR expression in NEPC makes this an interesting subgroup in regards of perspectives for GRPR-TRT. Among the 20 NEPC samples, 14 (70%) showed high GRPR expression, while only 3 (15%) were PSMA positive. Interestingly, all tumors with high PSMA expression also had high GRPR expression. Furthermore, among NEPC metastatic samples, 90% (9/10) were GRPR-high, as compared to only 10% (1/10) being PSMA-high (Fig. 3).

## Discussion

While GRPR-TRT has shown to be effective, e.g. in a seminal study by Kurth et al.^20^, the expression of GRPR in PCa, potentially influenced by molecular and clinical parameters, remains understudied. Accordingly, this study aimed to correlate clinical, biological, molecular and pathological characteristics, with GRPR expression in PCa PDX models, and in comparison to PSMA expression.

In the studied TMAs, 65% of the samples were CRPC, and 55% concerned metastatic disease. Interestingly, more than 70% of the samples had high GRPR expression, which is in contrast to previous reports^11,21^. However, PDX models, though retaining key features of the original tumor, do not fully reflect tumors in patients. This is illustrated by the number of samples having high Ki-67 expression (100%). Therefore, the high number of GRPR positive samples might not be representative for the clinical situation. Based on our previous experience, the cut-off used for IHC is frequently associated with high uptake of GRPR-targeting radiopharmaceuticals^12^, although it is not validated prospectively by means of GRPR-PET. Of note, the large number of samples included in the current study is clearly advantageous. Association with pathological parameters showed that the expression of GRPR is not lost during metastatic spread as there were no significant differences between primary tumors and metastatic samples. This finding confirms earlier reported studies^22^, including an autoradiography study demonstrating no differences in GRPR expression between match-paired androgen-independent primary and metastatic PCa lesions^23^, identifying GRPR as a relevant target for TRT of PCa, independent of the disease spread. Moreover, the tumor site also showed to have no influence on GRPR expression. This is in line with in vivo studies showing high uptake of the GRPR-targeting radiopharmaceuticals [^177^Lu]Lu-RM2 in bone metastases, metastatic lymph nodes and other visceral metastases^20^. Regarding disease progression, as expected, GRPR is highly expressed in early phase disease (androgen-naïve) with 16/19 (84%) of the samples being GRPR positive. Nevertheless, 10/13 (77%) of androgen sensitive samples, and 47/60 (78%) of CRPC samples were also GRPR positive. This finding confirms a recent study in which GRPR expression in advanced PCa showed to be associated with shorter PFS and higher prostate specific antigen (PSA) values, and suggests a role of GRPR in advanced PCa^13^. Another important finding is the high expression of GRPR in NEPC and its higher frequency of expression in this PCa type compared to PSMA expression. NEPC are dedifferentiated tumors that are difficult to follow-up and to characterize, as PSA values are frequently low and molecular features present in differentiated cells (AR, PSMA, PSA) can be lost^24,25^. Moreover, NEPC is rare at the time of initial diagnosis but much more common in patients treated with the combination of androgen deprivation therapy and AR pathway inhibitors, e.g. abiraterone and enzalutamide^26^. In the investigated series, 70% of NEPC samples come from patients under treatment. Neuropeptides, e.g. GRP or bombesin (ligand of the GRPR), are frequently secreted by tumor cells with neuroendocrine phenotype as alternative AR-independent growth signals^27^. High GRP expression in NEPC has been associated with early treatment failure^28^. In the studied NEPC samples, GRPR expression was higher than that of PSMA and, importantly, NEPC samples with high GRPR expression were mostly metastatic (90%). This finding might pave the way for a TRT approach for this specific subgroup of lethal PCa’s. Our finding is in line with a previous pilot study performed on 7 human NEPC samples in which all samples had high GRPR expression while PSMA expression was low^27^. Comparison of GRPR-targeting radionuclide imaging with that of [^18^F]-FDG, would be of great interest^29^. The 3MTPO study has recently shown that among patients with [^18^F]-FDG negative and [^68^Ga]Ga-PSMA negative PCa (likely to be NEPC), 16% of them had at least one [^68^Ga]Ga-DOTATATE positive, i.e. somatostatin receptor subtype 2 (SSTR2) positive, lesion^30^. Therefore, better characterization of NEPC clones with GRPR-PET would be useful. Currently, a clinical trial is ongoing recruiting patients with NEPC in which sequential PSMA-PET, then SSTR2-PET (if PSMA-PET is negative), then GRPR-PET (if SSTR2-PET is negative) will be performed (NCT06379217). Valuable data will be obtained from this trial for potential GRPR-targeting radionuclide imaging and therapy of this PCa subtype. Paired comparison of GRPR expression in NEPC samples with other relevant targets, including SST2R, DLL3, CDCP1, CD46, would also be valuable, as DLL3 is expressed in 78% of NEPC samples^31^, and CD46 and CDCP1 markers may be also overexpressed in NEPC^7,32^.

Association with molecular markers were next analyzed. As expected, GRPR was more often expressed in tumors harboring features associated with a good prognosis, such as AR-positivity, no loss of PTEN, and absence of vimentin (a marker of epithelial-mesenchymal transition when positive). Interestingly high GRPR expression was also found in more than 85% of PSMA positive tumors supporting the use of GRPR as a relevant target in CRPC, thus anticipating clinical impact of GRPR-TRT in metastatic CRPC patients as it is the case with PSMA-targeting radiopharmaceuticals. Unfortunately, the limited number of samples with regards to treatment history did not allow for robust association of GRPR expression with this parameter.

**Fig. 4:**
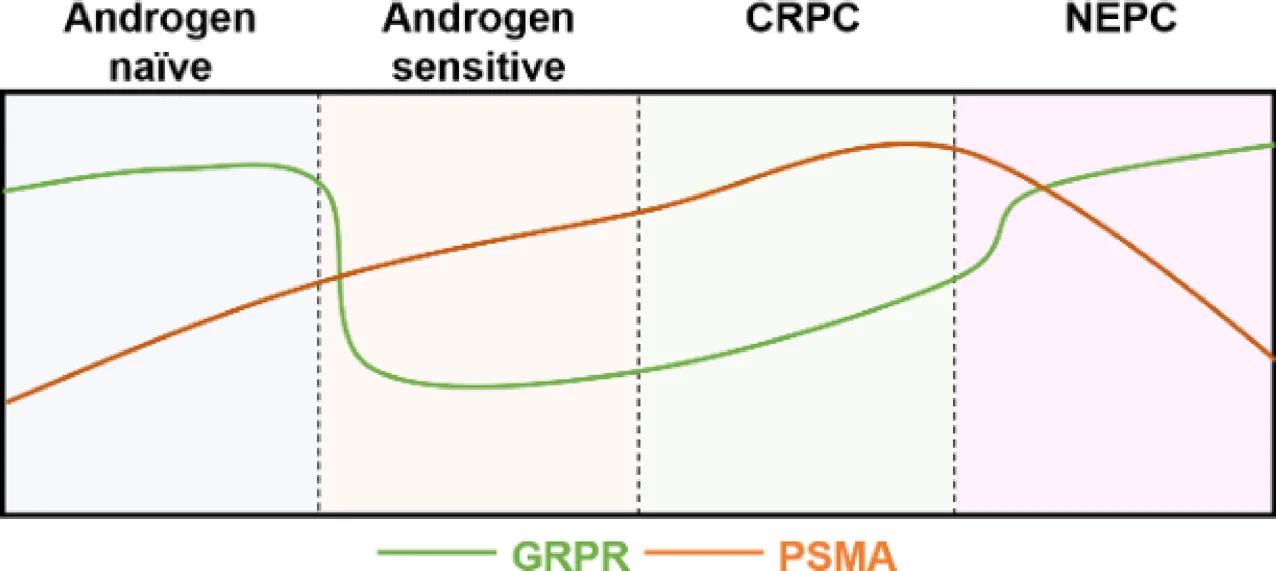
Proposed evolution of gastrin releasing peptide receptor (GRPR) and prostate specific membrane antigen (PSMA) expression across prostate cancer. CRPC=castration resistant prostate cancer, NEPC=neuroendocrine prostate cancer.

Considering the widespread use of PSMA-TRT and for a cost-effectiveness approach of GRPR-TRT, a PSMA-stratified analysis was performed. More than 50% of the PSMA negative samples have GRPR expression. Interestingly, in samples with low PSMA and low AR expression, GRPR expression was high in 43% of samples (Table 5). Recent data has shown that long-term anti-androgen treatment increases the secretion of GRP and its cognate receptor GRPR which in turn promotes the progression of PCa to castration resistance^27^. Thus, it seems that GRPR reappears in a divergent clonal neuroendocrine evolution of CRPC (Fig. 4).

In addition to molecular markers already associated with high GRPR expression, in the overall population, high GRPR expression in PSMA negative tumors was also more frequently observed in chromogranin positive tumors (a marker of neuroendocrine differentiation) and loss of Rb expression (a common observation in NEPC). It is interesting to note that, inactivation of Rb has shown to promote an aggressive NEPC phenotype^33^ which strengthens the role of GRPR in NEPC. Finally, the main predictor of high GRPR expression in PSMA negative tumors was the absence of PTEN loss in the multivariate analysis with an OR of 14.

Altogether, we have demonstrated that GRPR has two profiles of expression; the first one in early-stage disease and the second one in advanced late-stage disease, mainly in CRPC and NEPC, thus envisioning clinical impact of GRPR-TRT. In this context it is important to highlight the specific added-value of GRPR-targeting radiopharmaceuticals compared to PSMA-targeting radiopharmaceuticals. PSMA-targeting radiopharmaceuticals are associated with (severe) salivary gland toxicity which significantly impacts the quality of life of cancer patients, especially when cytotoxic alpha-emitting radionuclides , currently being studies, are applied^34,35^. GRPR-targeting radiopharmaceuticals, on the other hand, have shown to have a more favorable safety profile; despite GRPR expression in the healthy pancreas, no pancreatic toxicity is observed^20,36^. However, standard uptake values (SUV) values in PSMA-PET imaging are frequently higher than that of GRPR-PET which favors the use of PSMA-targeting radiopharmaceuticals. Nevertheless, GRPR-targeting radiopharmaceuticals would be a relevant alternative in case of weak response and/or toxicity^37^.

Moreover, with the aim of improving treatment efficacy, PSMA-TRT using radiopharmaceuticals coupled to radionuclides other than lutetium-177 (as is routinely clinically applied/approved) are being studied. Next to the aforementioned alpha-emitting radionuclides, this includes the beta-, Auger and conversion electron emitter terbium-161. Terbium-161 can mainly be advantageous for elimination of small cancer cell clusters, as the use of this radionuclide results in a higher tumor dose deposit which is safe in humans^38,39^. The PSMA-targeting radiopharmaceuticals applied are internalized vectors, resulting in close proximity of the radionuclide to the DNA allowing the Auger and conversion electrons to cause additional DNA damage and subsequent tumor cell death. However, the Auger and conversion electrons emitted by terbium-161 can also provide massive dose deposit when located at the cell membrane^40,41^ which is the case when linked to GRPR-targeting radiopharmaceuticals, which are mainly membrane-bound radiolabeled GRPR-antagonists^10^, and thus the use of terbium-161 can also provide additional clinical benefit in the case of GRPR-TRT.

This study also has limitations. For one, there is no validated GRPR score correlating to a positive GRPR-PET. Various scores have been proposed in literature with different outcomes^12,42–44^. Thus, there is a need for standardization of GRPR IHC. Additionally, we were not able to evaluate the impact of GRPR expression on patient prognosis. This should be evaluated in a separate study. Moreover, the proportion of PSMA negative tumors (49%) in the TMAs is quite high which indirectly favors GRPR. However, NEPC samples and low grade PCa, both frequently PSMA negative, represent ~ 35% of our cohort. Finally, results of GRPR expression in NEPC and PSMA negative samples are from small-size groups which limits generalization.

## Conclusion

In summary, we successfully analyzed GRPR expression on a dedicated set of TMAs mostly composed of advanced PCa PDX tissues using IHC. We demonstrated that GRPR has two profiles of expression; the first one in early-stage disease and the second one in advanced disease stages, mainly in CRPC and NEPC. The selective high GRPR expression in NEPC in contrast to the absence of PSMA expression, might open new avenues for GRPR-TRT in the competitive field of PSMA-TRT, when confirmed in larger studies.

## Supplementary Information

Below is the link to the electronic supplementary material.

Supplementary Material 1

## Data Availability

The data generated during this study can be obtained from the corresponding author upon reasonable request.
