# Supplementary material for: The multifaceted expression of the gastrin releasing peptide receptor in prostate cancer
Source: Sci Rep. 2026 Aug 12;16:37744. doi: 10.1038/s41598-026-64701-7 (PMC13754565; doi:10.1038/s41598-026-64701-7)
Supplement: Supplementary file 1 [file 41598_2026_64701_MOESM1_ESM.pdf]

MINISTÈRE
DE L'ENSEIGNEMENT
SUPÉRIEUR
ET DE LA RECHERCHE

Liberté
Égalité
Fraternité

Direction générale
de la recherche et de l'innovation

Service de la performance du financement et de la contractualisation avec les organismes de recherche

Département des pratiques de recherche réglementées

Cellule bioéthique
Affaire suivie par : Dorhôn MONDOUA

Mél : gestion.conservation@recherche.gouv.fr

1, rue Descartes
75231 Paris SP 05

Paris le 26/07/2023

Le ministère de l'enseignement supérieur, de la recherche et de l'innovation

Monsieur le Président

Université de Bordeaux

Objet : Conservation et préparation à des fins scientifiques d'éléments du corps humain pour les besoins de programmes de recherche (responsable scientifique :Sandrine BERTRAND)

Conformément aux dispositions des articles R. 1243-49 et suivants du code de la santé publique, vous avez adressé à nos services un dossier de déclaration d'activité de conservation et de préparation d'éléments du corps humain pour les besoins des programmes de recherche de l'organisme que vous représentez.

La direction générale de la recherche et de l'innovation du ministère (cellule de bioéthique) accuse réception de votre dossier, enregistré sous le **n°DC-2023-5565** (responsable scientifique : Sandrine BERTRAND).

En l'absence de décision expresse d'opposition de l'autorité administrative, l'activité déclarée peut commencer à l'issue d'un délai de deux mois à compter de la date de cet accusé de réception. Toutefois, ce délai peut être suspendu s'il est demandé de fournir des informations manquantes ou incomplètes ou des compléments nécessaires à l'expertise du dossier.

Dorhôn MONDOUA
Chargée d'affaires réglementaires
